# Comparison of the Association Between Arterial Stiffness Indices and Heart Failure in Patients With High Cardiovascular Risk: A Retrospective Study

**DOI:** 10.3389/fcvm.2021.782849

**Published:** 2021-11-15

**Authors:** Chan Joo Lee, Minjae Yoon, Jaehyung Ha, Jaewon Oh, Sungha Park, Sang-Hak Lee, Seok-Min Kang

**Affiliations:** Division of Cardiology, Severance Hospital, Yonsei University College of Medicine, Seoul, South Korea

**Keywords:** heart failure, arterial stiffness, central blood pressure, pulse wave velocity, retrospective study, prospective cohort, brachial pulse pressure

## Abstract

**Objective:** Study findings of the relationship of each arterial stiffness index with incident heart failure (HF) are conflicting. We aimed to compare the association between the indices of arterial stiffness and the risk of HF.

**Methods:** We analysed 3,034 patients from a prospective cohort that enrolled patients with high cardiovascular risk. They underwent brachial-ankle pulse wave velocity (baPWV), brachial pulse pressure (PP), carotid-femoral pulse wave velocity (cfPWV), and central PP measurements.

**Results:** Over a median follow-up of 4.7 years (interquartile range, 3.4–5.8 years), 65 HF events occurred. The incidence rate of HF was 4.7 per 1,000 person-years [95% confidence interval (CI), 3.7–6.0]. There was no difference in baPWV in those with and without HF events (1,561 ± 401 and 1,520 ± 321 cm/s, respectively, *P* = 0.415); however, there was a significant difference in brachial PP (63.2 ± 16.9 vs. 52.3 ± 11.5 mmHg, *P* < 0.001), cfPWV (11.0 ± 3.1 vs. 9.4 ± 2.4 m/s, *P* < 0.001) and central PP (56.6 ± 19.9 vs. 42.9 ± 13.8 mmHg, *P* < 0.001). In the multivariable-adjusted model, brachial PP [hazards ratio (HR) per standard deviation unit (SDU), 1.48; 95% CI, 1.19–1.84, *P* < 0.001], cfPWV (HR per SDU, 1.29; 95% CI, 1.02–1.63, *P* = 0.032) and central PP (HR per SDU, 1.44; 95% CI, 1.17–1.78; *P* < 0.001) were associated with incident HF, but baPWV was not (HR per SDU, 0.83; 95% CI, 0.63–1.10; *P* = 0.198). In the receiver operating characteristic analysis, the area under the curve (AUC) of brachial PP (*P* < 0.001), cfPWV (*P* = 0.003) or central PP (*P* = 0.001) was larger than that of baPWV, and there was no difference in the AUCs of brachial PP, cfPWV and central PP.

**Conclusion:** Among arterial stiffness indices, brachial PWV was less associated with the risk of heart failure, and brachial PP and measures representing central hemodynamics were highly associated with incident HF.

## Introduction

It is estimated that there are 65.3 million people with heart failure (HF) worldwide, and the prevalence of HF is increasing (1). The prognosis of HF is very poor, and the survival rate at 5 years after HF diagnosis is only 45.5% (2). Despite the development of medications and devices for HF, the risk of death owing to HF remains high. Therefore, there is a need for a pre-emptive prevention strategy that appropriately selects patients at high risk of incident HF and controls risk factors.

The hemodynamic burden on the heart contributes to HF development (3). The heart is an organ that produces blood flow; however, it is affected by the pressure within the aorta, which is called ventricular–arterial coupling (4). A rise in afterload increases myocardial oxygen demand and causes myocardial wall thickening, and this long-term change has a detrimental effect on the left ventricle (LV), leading to symptomatic HF (5). Afterload is affected by complex factors such as pulsatile blood flow and the properties of blood vessels. In particular, an aorta with reduced elasticity has a low capacity as a reservoir for the pulsatile pressure of ventricular ejection and increases afterload (4). Therefore, arterial stiffness, which is a general term for reduced arterial elasticity, showed an association with the development of HF in previous studies (6, 7).

Arterial stiffness can be expressed in several ways. Carotid-femoral pulse wave velocity (cfPWV) and brachial-ankle pulse wave velocity (baPWV) are representative non-invasive measures of arterial stiffness; they are related to the occurrence of cardiovascular disease (CVD) and can be utilised for cardiovascular risk assessment (8, 9). Another index of arterial stiffness is pulse pressure (PP). Like brachial PP, central PP is closely related to CVD (10). Among these measures, brachial PP and cfPWV have been investigated as a predictor for incident HF (6, 11) but studies on other measures are lacking. Therefore, this study aimed to compare the association between the indices of arterial stiffness and the risk of HF.

## Materials and Methods

### Ethics Statements

The Institutional Review Board of the Yonsei University Health System Clinical Trial Center approved the study protocol (4-2013-0581), and written informed consent was obtained from all participants.

### Study Design and Population

This retrospective study analysed participants of the Cardiovascular and Metabolic Disease Etiology Research Center-High Risk Cohort (CMERC-HI). Briefly, CMERC-HI is a prospective cohort study aimed at developing more specific preventive strategies for patients with a high risk of CVD (ClinicalTrials.gov ID: NCT02003781). The following patients were included in the cohort: high-risk patients with hypertension [estimated glomerular filtration rate (eGFR) > 60 mL/min/1.73 m^2^ with target organ damage or eGFR ≤ 60 mL/min/1.73 m^2^]; patients with diabetes mellitus with albuminuria; anuric patients with end-stage renal disease who were undergoing dialysis; relatives of patients with acute myocardial infarction (men aged <55 years; women aged <65 years); patients with asymptomatic atherosclerotic CVD [abdominal aorta diameter ≥ 3 cm or ankle-brachial index (ABI) <0.9, carotid plaque or carotid intima-media thickness ≥ 0.9 mm, asymptomatic old cerebrovascular accident, or >30% stenosis in at least one major coronary artery]; patients with rheumatoid arthritis aged > 40 years and taking methotrexate and a steroid; patients with atrial fibrillation with a CHA2DS2-VASc score ≥ 1; and kidney transplant recipients at >3 months after transplantation. Persons aged > 20 years who met at least one of the inclusion criteria were enrolled. The exclusion criteria were as follows: a history of acute coronary syndrome, symptomatic coronary artery disease or symptomatic peripheral artery disease, or HF; a life expectancy of <6 months; pregnancy; or a history of contrast allergy and related adverse effects.

### Central Hemodynamic Measurements, baPWV and Brachial Pulse Pressure

The central hemodynamic were evaluated using the SphygmoCor system (AtCor Medical, Sydney, Australia) with patients in the sitting position after 10 min of rest. A high-fidelity micromanometer (Millar Instruments, Houston, TX, USA) was used to record peripheral pressure waveforms from the radial arteries, as reported previously (12, 13). Radial artery waveforms were obtained from the patient's arm without an arteriovenous fistula. The SphygmoCor system obtains the ascending aortic pressure waveform from the radial artery waveform using its validated mathematical transfer function. The central systolic blood pressure (BP), diastolic BP, PP, augmentation pressure, forward wave amplitude, and augmentation index were acquired from the aortic pressure waveform analyses. Central PP was calculated as the difference between the systolic and diastolic BPs. cfPWV was measured as specified previously (14). Briefly, electrocardiogram and carotid/femoral pulse waves were obtained simultaneously to calculate the transit time using the foot-to-foot method. The distance travelled by the pulse wave was calculated by subtracting the sternal notch-right carotid site from the right femoral site-sternal notch distances (14).

baPWV was measured using a volume-plethysmography device (OMRON, Tokyo, Japan). The patients were examined while resting in the supine position. Electrocardiographic electrodes were placed on both wrists, and cuffs were wrapped on both arms and ankles. Pulse volume waveforms at both brachial and posterior tibial arteries were recorded using a semiconductor pressure sensor after patients rested for at least 5 min. The heart rate was continuously recorded with flow and pressure tracings gated to the electrocardiogram, and baPWV was calculated automatically using time-phase analysis. The distance between the upper arm and ankle was estimated based on height. We used the average baPWV from right and left measurements in the analysis. Brachial pulse pressure was calculated using the systolic and diastolic blood pressures derived during baPWV measurement.

### Assessment of Heart Failure Outcomes

An HF event was identified by hospitalisations related to HF symptoms. HF diagnoses were adjudicated by a three-physician committee after extensive review of inpatient medical records using the following clinical criteria modified from the European Society of Cardiology definition (15): (1) HF with reduced ejection fraction (HFrEF) was defined as left ventricular ejection fraction (LVEF) ≤ 40% on imaging study (echocardiography, technetium-99m sestamibi myocardial imaging, or cardiac magnetic resonance imaging) and plasma N-terminal pro-B-type natriuretic peptide (NT-proBNP) level ≥ 600 pg/mL and (2) HF with preserved ejection fraction (HFpEF) was defined as LVEF > 40% and relaxation abnormality of the LV filling pattern on echocardiography and NT-proBNP level ≥ 300 pg/mL. The date of onset was noted as the first date of hospitalisation owing to HFrEF or HFpEF.

### Statistical Analysis

We divided study participants into two groups according to incident HF status. We used the *t*-test and chi-square test to compare continuous and categorical variables, respectively, between the two groups. Continuous variables are expressed as mean ± standard deviation, and categorical variables are expressed as *n* (%).

Receiver operating characteristic (ROC) analysis was performed to identify the best cut-off values and assess the performance of baPWV, brachial PP, cfPWV, and central PP for predicting incident HF. We calculated the areas under the ROC curve (AUCs) for baPWV, brachial PP, cfPWV, and central PP and used the method of DeLong et al. to test the statistical significance of the differences between them (16). We defined the high-risk category as a value higher than the cut-off values of baPWV, brachial PP, cfPWV, and central PP. Kaplan-Meier survival curve analysis was used to assess the cumulative rate of incident HF according to high-risk categories based on baPWV, brachial PP, cfPWV, and central PP values. We used Cox proportional hazards regression analysis to assess the associations between categorical (high-risk categories) and continuous measures of arterial stiffness and incident HF using the univariable model, age- and sex-adjusted model, and multivariable model adjusted for age, sex, body mass index, antihypertensive medication usage, hemoglobin level, and eGFR. Statistical analyses were conducted using R software, version 4.0.4 (R Foundation for Statistical Computing, Vienna, Austria), assuming a threshold of significance at *P* < 0.05.

## Results

In total, 3,270 participants were enrolled from November 2013 to June 2018 at the Severance Hospital in Seoul, Republic of Korea. Among the participants, 236 did not undergo central hemodynamic measurements or baPWV measurement. Finally, 3,034 participants were included in the final analyses.

Over a median follow-up of 4.7 years (interquartile range, 3.4–5.8 years), 65 HF events occurred, of which 11 were HFrEF and 54 were HFpEF. The incidence rate of HF was 4.7 [95% confidence interval (CI), 3.7–6.0] per 1,000 person-years. Table 1 shows the baseline characteristics of all study participants. Among 3,034 participants, the average age was 59.2 ± 11.6 years, 54.6% of the participants were men, and 83.5 and 45.5% had hypertension and diabetes, respectively. Participants with incident HF had higher systolic BP than those without incident HF, despite a higher rate of antihypertensive drug use. Based on the laboratory findings, we found that participants with incident HF had lower levels of hemoglobin and high-density lipoprotein cholesterol and lower eGFR than those without.

**Table 1:** The clinical characteristics of study participants according to incident heart failure.

	**Total (*N* = 3,034)**	**No incident HF (*N* = 2,969)**	**Incident HF (*N* = 65)**	***P*-value**
Age, years	59.2 ± 11.6	59.1 ± 11.6	61.4 ± 12.3	0.110
Men, *N* (%)	1,656 (54.6)	1,618 (54.5)	38 (58.5)	0.611
BMI, kg/m^2^	25.1 ± 3.6	25.1 ± 3.6	24.7 ± 3.2	0.390
Hypertension, *N* (%)	2,534 (83.5)	2,474 (83.3)	60 (92.3)	0.155
Type 2 diabetes, *N* (%)	1,381 (45.5)	1,346 (45.4)	35 (53.8)	0.217
SBP, mmHg	128.3 ± 18.0	128.0 ± 17.7	141.3 ± 25.6	<0.001
DBP, mmHg	76.7 ± 10.7	76.8 ± 10.6	74.8 ± 14.2	0.285
Antihypertensive medications, *N* (%)	2,508 (82.7)	2,446 (82.4)	62 (95.4)	0.010
RASB, *N* (%)	1,935 (63.8)	1,891 (63.7)	44 (67.7)	0.594
CCB, *N* (%)	1,507 (49.7)	1,462 (49.3)	45 (69.2)	0.002
BB, *N* (%)	807 (26.6)	770 (25.9)	37 (56.9)	<0.001
Diuretics, *N* (%)	755 (24.9)	718 (24.2)	37 (56.9)	<0.001
**Laboratory**				
Hemoglobin, g/dL	13.3 ± 2.0	13.4 ± 2.0	11.5 ± 2.1	<0.001
TC, mg/dL	173.4 ± 40.2	173.3 ± 38.6	179.6 ± 85.4	0.558
HDL-C, mg/dL	49.5 ± 13.7	49.6 ± 13.6	42.2 ± 15.1	<0.001
LDL-C, mg/dL	95.2 ± 33.1	95.0 ± 32.0	103.8 ± 66.5	0.314
TG, mg/dL	141.0 ± 91.0	141.2 ± 91.4	131.6 ± 72.1	0.315
eGFR, mL/min/1.73 m^2^	68.5 ± 34.3	69.4 ± 33.8	28.8 ± 30.8	<0.001

*HF, heart failure; BMI, body mass index; SBP, systolic blood pressure; DBP, diastolic blood pressure; RASB, renin angiotensin system blockers; CCB, calcium channel blockers; BB, beta blockers; TC, total cholesterol; HDL-C, high-density lipoprotein cholesterol; LDL-C, low-density lipoprotein cholesterol; TG, triglyceride; BUN, blood urea nitrogen; eGFR, estimated glomerular filtration rate*.

**Figure 1:**
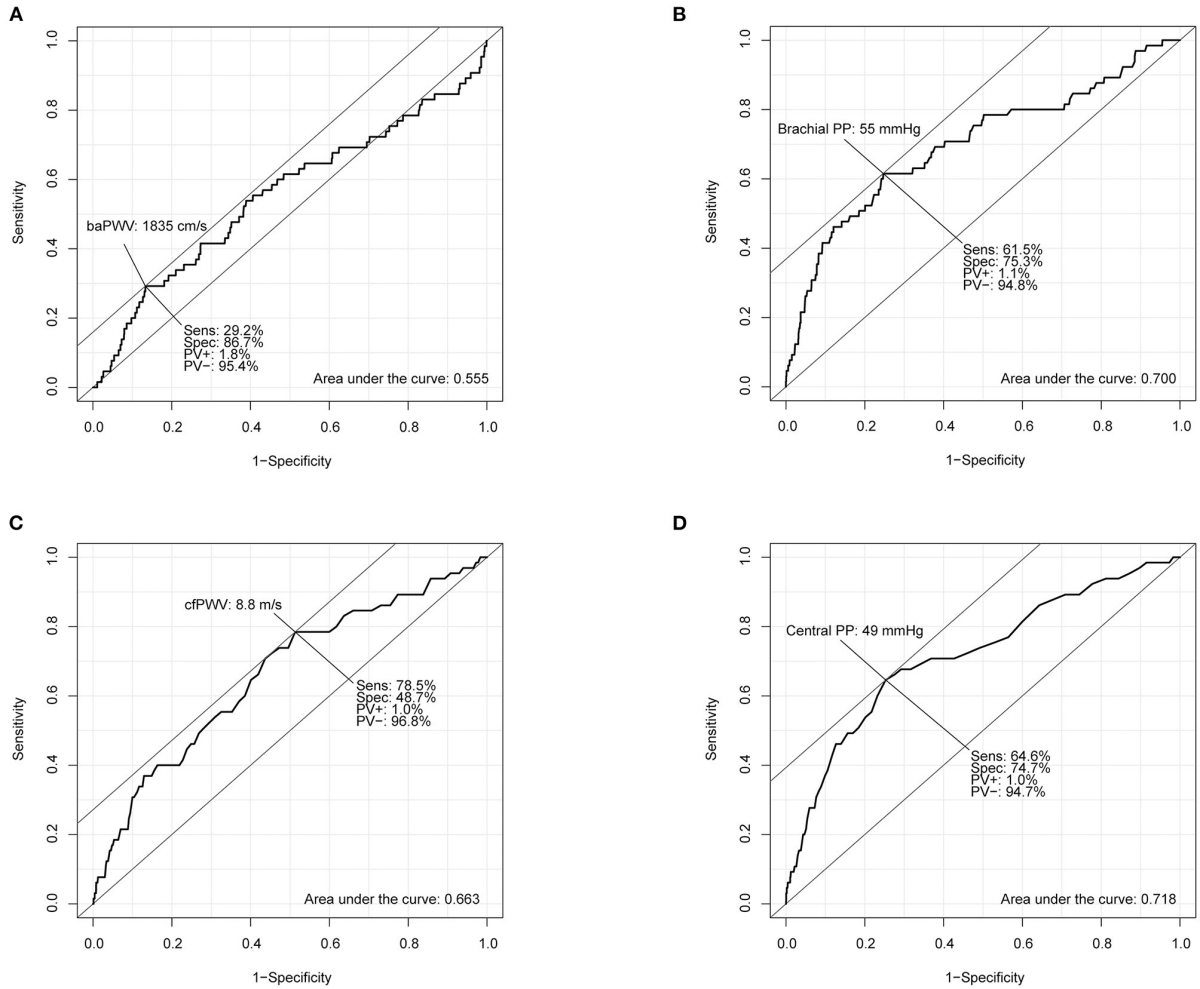
Receiver operating characteristic curve analyses of brachial-ankle pulse wave velocity (baPWV; **A**), brachial pulse pressure (brachial PP; **B**), carotid-femoral pulse wave velocity (cfPWV; **C**), and central pulse pressure (central PP; **D**) for predicting incident heart failure.

Results of the ROC curve analysis of the association between arterial stiffness measures and incident HF are shown in Figure 1. According to the ROC curve of baPWV, the AUC was 0.555 (95% CI, 0.474–0.636) and best cut-off value was 1,835 cm/s for prediction of incident HF. However, the AUCs of brachial PP, cfPWV and central PP were 0.700 (95% CI, 0.625–0.774), 0.663 (95% CI, 0.593–0.733) and 0.718 (95% CI, 0.650–0.787), respectively. The AUC of baPWV (*P* < 0.001), cfPWV (*P* = 0.003) or central PP (*P* < 0.001) was larger than that of baPWV. There was no difference in the AUCs between brachial PP and cfPWV (*P* = 0.292), the AUCs between brachial PP and central PP (*P* = 0.445), and the AUCs between brachial PP and central PP (*P* = 0.145). The ROC curve analysis revealed that brachial PP of 55 mmHg, cfPWV of 8.8 m/s and central PP of 49 mmHg were cut-off values with the highest sensitivity and specificity for predicting incident HF.

Table 2 presents the arterial stiffness measures and central hemodynamic parameters according to incident HF. Although there was no difference in baPWV between the two groups, the proportion of participants who had baPWV ≥ 1,835 cm/s was higher among those with incident HF than among those without incident HF. In contrast, brachial PP was significantly different between two groups. There a higher proportion of participants with brachial PP ≥ 55 mmHg among participants with incident HF than among those without HF.

**Table 2:** The arterial stiffness measures and central hemodynamic parameters according to incident heart failure.

	**Total (*N* = 3,034)**	**No incident HF (*N* = 2,969)**	**Incident HF (*N* = 65)**	***P*-value**
baPWV, cm/s	1,521.1 ± 322.9	1,520.2 ± 321.0	1,561.3 ± 401.0	0.415
baPWV ≥ 1,835 cm/s, *N* (%)	480 (15.8)	396 (13.3)	19 (29.2)	<0.001
Brachial PP, mmHg	52.6 ± 11.7	52.3 ± 11.5	63.2 ± 16.9	<0.001
Brachial PP ≥ 55 mmHg, N (%)	1,126 (37.1)	1,083 (36.5)	43 (66.2)	<0.001
cfPWV, m/s	9.5 ± 2.4	9.4 ± 2.4	11.0 ± 3.1	<0.001
cfPWV ≥ 8.8 m/s, *N* (%)	1,002 (33.0)	1,581 (53.3)	51 (78.5)	<0.001
Central SBP, mmHg	118.8 ± 18.9	118.5 ± 18.6	132.8 ± 25.5	<0.001
Central DBP, mmHg	75.6 ± 10.5	75.6 ± 10.5	76.2 ± 12.7	0.704
Augmentation index	27.1 ± 12.6	27.0 ± 12.6	29.8 ± 11.7	0.079
Central PP, mmHg	43.2 ± 14.1	42.9 ± 13.8	56.6 ± 19.9	<0.001
Central PP ≥ 49 mmHg, N (%)	793 (26.1)	818 (27.6)	43 (66.2)	<0.001

*HF, heart failure; baPWV, brachial ankle pulse wave velocity; cfPWV, carotid femoral pulse wave velocity; PP, pulse pressure*.

In terms of central hemodynamic parameters, cfPWV was significantly higher in participants with incident HF than in those without incident HF. The proportion of participants with cfPWV ≥ 8.8 m/s was also higher among those with incident HF than among those without incident HF. Additionally, participants with incident HF had higher central PP than those without incident HF. There was a higher proportion of participants with central PP ≥ 49 mmHg among participants with incident HF than among those without HF.

**Figure 2:**
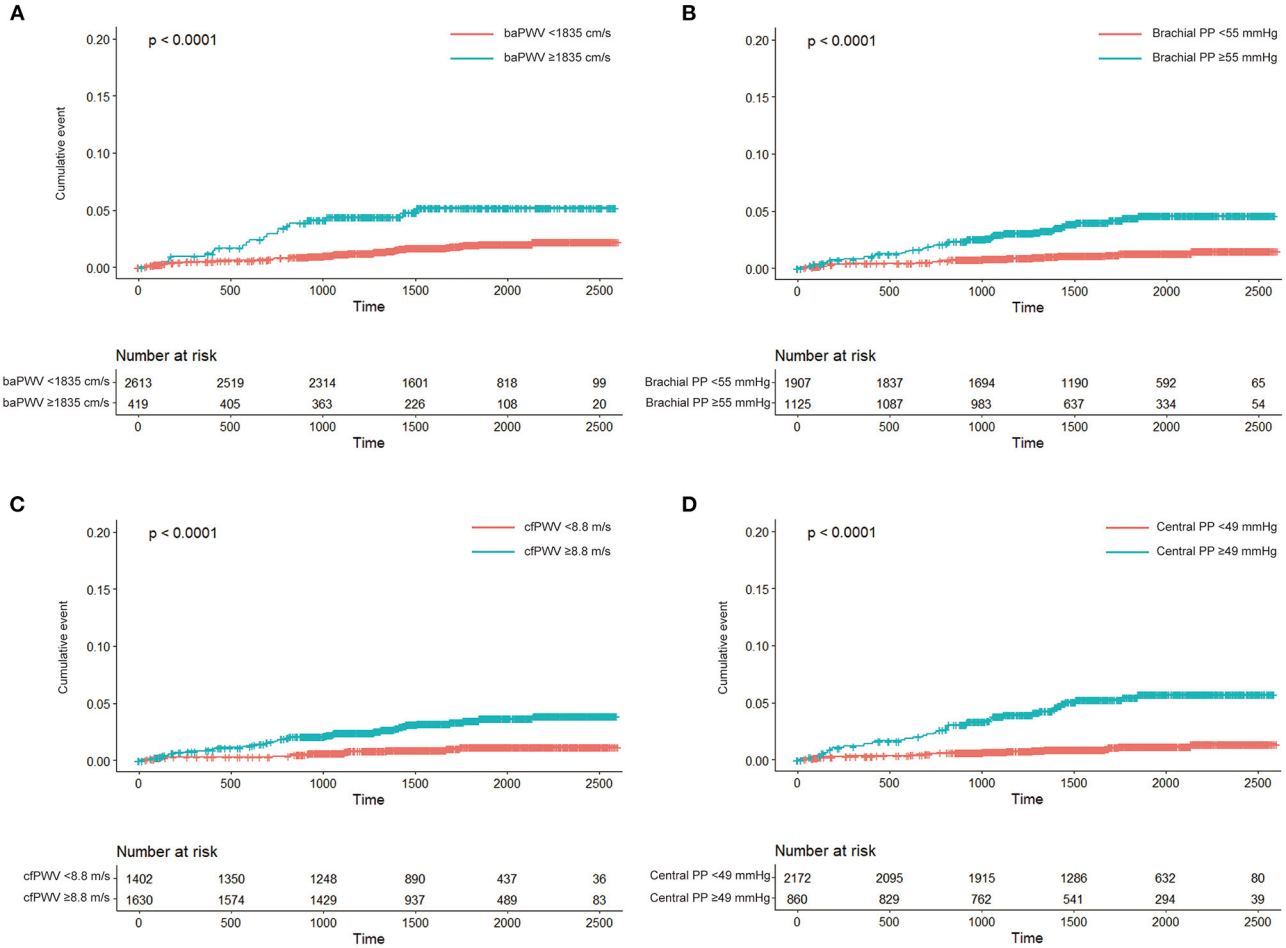
Kaplan-Meier curve analyses of cumulative incidence of heart failure according to high-risk category of brachial-ankle pulse wave velocity (baPWV; **A**), brachial pulse pressure (brachial PP; **B**), carotid-femoral pulse wave velocity (cfPWV; **C**), and central pulse pressure (central PP; **D**).

Participants with high-risk category of arterial stiffness were older, had higher rates of diabetes, higher blood pressure, and more reduced renal function than participant with low-risk category (Supplementary Tables 1–4). We analysed the associations of high-risk categories of arterial stiffness with incident HF. In the unadjusted analyses, baPWV ≥ 1,835 cm/s, brachial PP ≥ 55 mmHg, cfPWV ≥ 8.8 m/s, and central PP ≥ 49 mmHg were associated with the risk of incident HF (all, *P* < 0.05; Figure 2; Table 3). In the age- and sex-adjustment model, baPWV ≥ 1,835 cm/s, brachial PP ≥ 55 mmHg, cfPWV ≥ 8.8 m/s, and higher central PP ≥ 49 mmHg were associated with incident HF (all, *P* < 0.05; Table 3). There was significant association of cfPWV ≥ 8.8 m/s and central PP ≥ 49 mmHg with incident HF even after adjusting for additional covariates, except baPWV ≥ 1,835 cm/s and brachial PP ≥ 55 mmHg (Table 3).

**Table 3:** The association between arterial stiffness measures and incident heart failure.

	**Univariable model**		**Age- and sex-adjusted**		**Multivariable model**	
	**HR (95% CI)**	***P*-value**	**HR (95% CI)**	***P*-value**	**HR (95% CI)**	***P*-value**
**Categorical variables**						
baPWV ≥ 1,835 cm/s	2.69 (1.57–4.59)	<0.001	2.54 (1.44–4.48)	0.001	1.48 (0.82–2.67)	0.197
Brachial PP ≥ 55 mmHg	3.39 (2.03–5.67)	<0.001	3.43 (2.02–5.82)	<0.001	1.73 (0.95–3.14)	0.073
cfPWV ≥ 8.8 m/s	3.21 (1.78–5.80)	<0.001	3.18 (1.70–5.93)	<0.001	2.01 (1.02–3.95)	0.043
Central PP ≥ 49 mmHg	4.86 (2.91–8.13)	<0.001	5.15 (2.30–8.85)	<0.001	3.07 (1.68–5.64)	<0.001
Continuous variables^*^						
baPWV, cm/s	1.13 (0.90–1.43)	0.293	1.06 (0.82–1.37)	0.644	0.83 (0.63–1.10)	0.198
Brachial PP, mmHg	1.92 (1.62–2.28)	<0.001	1.93 (1.62–2.29)	<0.001	1.48 (1.19–1.84)	<0.001
cfPWV, m/s	1.62 (1.36–1.93)	<0.001	1.63 (1.35–1.97)	<0.001	1.29 (1.02–1.63)	0.032
Central PP, mmHg	1.85 (1.57–2.17)	<0.001	1.88 (1.59–2.22)	<0.001	1.44 (1.17–1.78)	<0.001

* *HR for incident HF expressed per standard deviation increment in each measure. SD for each measure were as follows: baPWV SD = 322.9 cm/s, brachial PP SD = 11.7 mmHg, cfPWV SD = 2.4 m/s, central PP SD = 14.1 mmHg*.

*Multivariable model adjusted for age, sex, body mass index, diabetes, mean blood pressure, hypertensive medication usage, hemoglobin level, and estimated glomerular filtration rate*.

*HR, hazard ratio; CI, confidence interval; baPWV, brachial ankle pulse wave velocity; cfPWV, carotid femoral pulse wave velocity; PP, pulse pressure; SD, standard deviation*.

We analysed the association between arterial stiffness measures as continuous variables and incident HF. In the unadjusted models, brachial PP, cfPWV and central PP were significantly associated with the risk of incident HF, but baPWV was not. These associations were slightly attenuated but remained in the age- and sex-adjustment model and in the additional adjustment model (Table 3). Additionally, we performed a sensitivity analysis in participants with ABI > 0.9 because baPWV may not be reliable in participants with significant peripheral artery stenosis. In this analysis, baPWV was not associated with the risk of HF, and cfPWV showed a weak association (Supplementary Table 5).

## Discussion

In this study, brachial PP, cfPWV and central PP were higher in those with incident HF than in those without incident HF, but baPWV was not different between the two groups. However, there were more participants with high-risk category of each arterial stiffness measure among those with incident HF than among those without incident HF. When several covariates were adjusted, cfPWV ≥ 8.8 m/s and central PP ≥ 49 mmHg were significantly associated with incident HF. In previous studies, baPWV > 1,800 cm/s, brachial PP ≥ 55 mmHg, cfPWV > 10 m/s, and central PP > 50 mmHg were defined as high risk for cardiovascular events (10, 17–19), and our results were not markedly different from them. As a continuous variable for each index, the risk of incident HF increased as brachial PP, cfPWV and central PP increased, except for baPWV. Therefore, through this study, we revealed that arterial stiffness is an independent risk factor for HF, and among the indices of arterial stiffness, measures representing central hemodynamics and brachial PP were more relevant for incident HF than baPWV.

cfPWV has been studied considerably in Europe and the United States, and a significant amount of clinical data have been accumulated; thus, it is considered a gold-standard measurement of arterial stiffness (20). In contrast, baPWV has been mainly studied in Asia, especially Japan (21). Although baPWV lacks large-scale and long-term study data compared to cfPWV, it has been extensively studied recently. The two measures differ methodologically. In the case of cfPWV, the carotid and femoral artery pulses are measured using tonometry and calculated using the time difference between the two pulses. In contrast, for baPWV, the pulses of the brachial artery and the ankle artery are measured, and the time difference between the two pulses is used to calculate pulse wave velocity (PWV). Therefore, cfPWV is considered to represent aortic stiffness, whereas baPWV represents both aortic stiffness and peripheral arterial stiffness (22). In a community-based cohort study of 2,287 patients, cfPWV and baPWV showed a strong positive association, and the two indices were nearly equivalent for predicting the presence of coronary artery disease and stroke (22). Nevertheless, the relationship between HF and arterial stiffness has been studied for only cfPWV.

However, a recent study on the relationship of baPWV with LV remodelling and diastolic dysfunction was published. In 202 untreated hypertensive patients, baPWV was significantly associated with parameters of LV remodelling and diastolic function, and it predicted diastolic dysfunction well (23). Although this study did not examine the association of baPWV with HF, it suggests that baPWV contributes to the development of HF, as LV remodelling and diastolic dysfunction in hypertensive patients are considered to be the first steps leading to HF.

Our study showed that indices such as cfPWV and central PP related to central hemodynamics were more closely related to HF than baPWV. This is probably because cfPWV or central PP is a representative index that better represents the hemodynamic burden on the LV in the pathophysiologic aspect of the development of HF. In a small study, central systolic BP and central PP were more strongly associated with LV diastolic dysfunction than baPWV (24).

Study findings on the relationship between central hemodynamic parameters and incident HF are conflicting (6, 7, 25). In Connie et al.'s study, both cfPWV and central PP were associated with incident HF in the age- and sex-adjusted model, but there was no statistical significance in the association between central PP and incident HF in a multivariable model that included several cardiovascular risk factors (6). In contrast, in our study, the association between central PP and incident HF was clear even in the model adjusted for multiple covariates. Connie et al. analysed the Framingham cohort study sample, whereas the cohort used in our study was hospital-based and had a relatively higher risk of CVD than the Framingham cohort. This can be supported by the fact that our study cohort had a higher rate of antihypertensive drug use and a higher prevalence of diabetes, although their age was younger than that of the Framingham cohort.

It is unclear whether central PP or cfPWV is more predictive of the occurrence of CVD. Although PP is considered a surrogate marker of arterial stiffness, aortic PWV and peripheral pressure wave reflection are the two main determinants of central PP. Although one study showed that both central PP and cfPWV were associated with renal microvascular damage, in a model that considered both, only central PP was an independent factor for determining changes in renal hemodynamics (26). There is often a mismatch between PP and PWV, and the Framingham offspring cohort analysed the relative contribution of PP and PWV to CVD. Patients with high central PP and high cfPWV had the highest cardiovascular risk. However, cfPWV was more likely to be involved in left ventricular hypertrophy and incident CVD than central PP (27). In our study, central PP showed a significant association with the risk of HF in both the categorical variable analysis model and the continuous variable analysis model. In contrast, cfPWV showed an association in the continuous variable model. Since there was no difference in the AUC of the ROC curves for incident HF in cfPWV and central PP, it is difficult to conclude which of the two indices is superior for predicting HF even in our study.

Brachial PP is the simplest measure of arterial stiffness. The association between brachial PP and the risk of HF has been known in the elderly population (11). In our study, brachial PP showed a close relationship with incident HF, and the AUC value for incident HF in brachial PP was comparable to that of cfPWV or central PP. Although brachial PP and baPWV are highly correlated indices (28), it was confirmed through this study that brachial PP is a superior index for predicting heart failure compared to baPWV. It may be because PP well reflects the afterload along with arterial stiffness. Since central aorta is located close to the target organ, it is thought that central PP has a stronger effect on the target organ damage and the development of cardiovascular disease than brachial PP (29). However, in our study, brachial PP was associated with the risk of HF to a degree very similar to that of central PP. This may be due to the characteristics of our study participants. Pressure amplification (peripheral/central pulse pressure ratio) is high in younger people, but pressure amplification is reduced in older people or people with advanced arterial stiffness (30).

This study had some limitations. First, since this cohort mainly included hypertensive or diabetic patients with target organ damage, and asymptomatic atherosclerotic CVD, the risk of cardiovascular disease was higher than that of general population (31, 32). The 10-year atherosclerotic CVD risk calculated by the pooled cohort equation was 15% on average. Therefore, the results of our study must be cautiously applied to the general population. In particular, most of those who progressed to HF had poor renal function at baseline. It seems to be natural because chronic kidney disease is an independent risk factor for HF (33). Nevertheless, this study showed that cfPWV and central PP were associated with the development of HF in the multivariable model including renal function, which is similar to previous findings (7). Second, despite the follow-up period of ~4 years, the number of individuals who developed HF was smaller than expected. This may be because we limited incident HF events to only HF hospitalisation to clarify the adjudication through the retrospective chart review. Heart failure is a clinical syndrome and its definition is ambiguous and not standardised. Therefore, we used modified criteria with an increased cut-off for NT-proBNP levels in the European Society of Cardiology definition (15). Because the NT-proBNP level supporting hospitalisation for heart failure is presented as 300 pg/mL in the European Society of Cardiology position paper (34). Third, as this hospital-based cohort study recruited patients from a single tertiary centre, most of the study individuals regularly visited outpatient clinics and were being managed for underlying cardiovascular risk factors. Although this study could not reflect the natural relationship between arterial stiffness measures and incident HF, its results are valuable in real-world clinical practise and applicable to patients with high cardiovascular risk.

In conclusion, among arterial stiffness indices, brachial PP, cfPWV, and central PP were better predictors of HF than baPWV. Brachial PP or central hemodynamic measures may help in risk stratification for the development of HF in patients at high cardiovascular risk.

## Data Availability Statement

The raw data supporting the conclusions of this article will be made available by the authors, without undue reservation.

## Ethics Statement

The studies involving human participants were reviewed and approved by Yonsei University Health System Clinical Trial Center. The patients/participants provided their written informed consent to participate in this study.

## Author Contributions

CJL: conceptualisation, funding acquisition, and writing—original draught. MY, JH, and SP: data curation. SP, S-HL, and S-MK: supervision. JO and S-MK: validation. S-MK: writing—review and editing. All authors contributed to the article and approved the submitted version.

## Funding

This work was supported by the National Research Foundation of Korea grant funded by the Korean government (MSIT) (Grant Number 2020R1C1C1013627).

## Conflict of Interest

The authors declare that the research was conducted in the absence of any commercial or financial relationships that could be construed as a potential conflict of interest.

## Publisher's Note

All claims expressed in this article are solely those of the authors and do not necessarily represent those of their affiliated organizations, or those of the publisher, the editors and the reviewers. Any product that may be evaluated in this article, or claim that may be made by its manufacturer, is not guaranteed or endorsed by the publisher.
