# Supplementary material for: Comparison of the Association Between Arterial Stiffness Indices and Heart Failure in Patients With High Cardiovascular Risk: A Retrospective Study
Source: Front Cardiovasc Med. 2021 Nov 15;8:782849. doi: 10.3389/fcvm.2021.782849 (PMC8634721; doi:10.3389/fcvm.2021.782849)
Supplement: Supplementary file 1 [file Data_Sheet_1.docx]

**SUPPLEMENTAL MATERIALS**

Supplemental Table 1. The clinical characteristics of study participants according to categories of baPWV

|  | baPWV <1835 cm/s  (n=2619) | baPWV ≥1835 cm/s  (n=415) | P value |
| --- | --- | --- | --- |
| Age, years | 58.0 ± 11.5 | 66.8 ± 8.8 | <0.001 |
| Men, N(%) | 1446 (55.2) | 210 (50.6) | 0.089 |
| BMI, kg/m^2^ | 25.1 ± 3.7 | 24.7 ± 3.5 | 0.037 |
| Hypertension, N(%) | 2171 (82.9) | 363 (87.5) | 0.063 |
| Type 2 diabetes, N(%) | 1094 (41.8) | 287 (69.2) | <0.001 |
| SBP, mmHg | 125.9 ± 16.3 | 142.9 ± 20.9 | <0.001 |
| DBP, mmHg | 76.5 ± 10.4 | 77.8 ± 12.2 | 0.046 |
| Antihypertensive medications, N(%) | 2152 (82.2) | 356 (85.8) | 0.082 |
| RASB, N(%) | 1660 (63.4) | 275 (66.3) | 0.028 |
| CCB, N(%) | 1266 (48.4) | 241 (58.1) | <0.001 |
| BB, N(%) | 681 (26.0) | 126 (30.4) | 0.071 |
| Diuretics, N(%) | 631 (24.1) | 124 (29.9) | 0.013 |
| Laboratory |  |  |  |
| Hemoglobin, g/dL | 13.4 ± 2.0 | 12.5 ± 2.0 | <0.001 |
| TC, mg/dL | 174.0 ± 39.8 | 169.8 ± 42.9 | 0.063 |
| HDL-C, mg/dL | 49.7 ± 13.7 | 47.9 ± 13.4 | 0.014 |
| LDL-C, mg/dL | 95.8 ± 32.5 | 91.5 ± 36.3 | 0.033 |
| TG, mg/dL | 140.9 ± 93.6 | 141.7 ± 73.4 | 0.844 |
| eGFR, mL/min/1.73 m^2^ | 70.4 ± 33.8 | 56.3 ± 34.4 | <0.001 |
| baPWV, brachial ankle pulse wave velocity; BMI, body mass index; SBP, systolic blood pressure; DBP, diastolic blood pressure; RASB, renin angiotensin system blockers; CCB, calcium channel blockers; BB, beta blockers; TC, total cholesterol; HDL-C, high-density lipoprotein cholesterol; LDL-C, low-density lipoprotein cholesterol; TG, triglyceride; BUN, blood urea nitrogen; eGFR, estimated glomerular filtration rate. | | | |

Supplemental Table 2. The clinical characteristics of study participants according to categories of brachial pulse pressure

|  | Brachial PP <55 mmHg  (n=1907) | Brachial PP ≥55 mmHg  (n=1126) | P value |
| --- | --- | --- | --- |
| Age, years | 57.2 ± 11.3 | 62.6 ± 11.2 | <0.001 |
| Men, N(%) | 1125 (59.0) | 531 (47.2) | <0.001 |
| BMI, kg/m^2^ | 24.9 ± 3.5 | 25.3 ± 3.8 | 0.011 |
| Hypertension, N(%) | 1560 (81.8) | 975 (86.6) | 0.001 |
| Type 2 diabetes, N(%) | 716 (37.6) | 665 (59.1) | <0.001 |
| SBP, mmHg | 121.6 ± 13.5 | 139.5 ± 19.1 | <0.001 |
| DBP, mmHg | 76.6 ± 10.0 | 76.9 ± 11.7 | 0.462 |
| Antihypertensive medications, N(%) | 1536 (80.5) | 971 (86.2) | <0.001 |
| RASB, N(%) | 1210 (63.5) | 724 (64.3) | 0.667 |
| CCB, N(%) | 862 (45.2) | 645 (57.3) | <0.001 |
| BB, N(%) | 415 (21.8) | 391 (34.7) | <0.001 |
| Diuretics, N(%) | 418 (21.9) | 336 (29.8) | <0.001 |
| Laboratory |  |  |  |
| Hemoglobin, g/dL | 13.8 ± 1.9 | 12.6 ± 2.0 | <0.001 |
| TC, mg/dL | 176.1 ± 41.4 | 169.0 ± 37.7 | <0.001 |
| HDL-C, mg/dL | 50.4 ± 13.8 | 48.0 ± 13.4 | <0.001 |
| LDL-C, mg/dL | 97.4 ± 34.3 | 91.4 ± 30.6 | <0.001 |
| TG, mg/dL | 143.1 ± 98.1 | 137.6 ± 77.7 | 0.101 |
| eGFR, mL/min/1.73 m^2^ | 73.5 ± 31.8 | 59.9 ± 36.5 | <0.001 |
| PP, pulse pressure; BMI, body mass index; SBP, systolic blood pressure; DBP, diastolic blood pressure; RASB, renin angiotensin system blockers; CCB, calcium channel blockers; BB, beta blockers; TC, total cholesterol; HDL-C, high-density lipoprotein cholesterol; LDL-C, low-density lipoprotein cholesterol; TG, triglyceride; BUN, blood urea nitrogen; eGFR, estimated glomerular filtration rate. | | | |

Supplemental Table 3. The clinical characteristics of study participants according to categories of cfPWV

|  | cfPWV <8.8 cm/s  (n=1402) | cfPWV ≥8.8 cm/s  (n=1632) | P value |
| --- | --- | --- | --- |
| Age, years | 54.6 ± 11.5 | 63.1 ± 10.1 | <0.001 |
| Men, N(%) | 754 (53.8) | 902 (55.3) | 0.433 |
| BMI, kg/m^2^ | 24.6 ± 3.6 | 25.5 ± 3.6 | <0.001 |
| Hypertension, N(%) | 1143 (81.5) | 1391 (85.2) | 0.014 |
| Type 2 diabetes, N(%) | 424 (30.3) | 957 (58.6) | <0.001 |
| SBP, mmHg | 121.6 ± 14.6 | 134.0 ± 18.7 | <0.001 |
| DBP, mmHg | 76.3 ± 10.1 | 77.1 ± 11.1 | 0.044 |
| Antihypertensive medications, N(%) | 1115 (79.5) | 1393 (85.4) | <0.001 |
| RASB, N(%) | 863 (61.6) | 1072 (65.7) | 0.020 |
| CCB, N(%) | 599 (42.8) | 908 (55.6) | <0.001 |
| BB, N(%) | 318 (22.7) | 489 (30.0) | <0.001 |
| Diuretics, N(%) | 277 (19.8) | 478 (29.3) | <0.001 |
| Laboratory |  |  |  |
| Hemoglobin, g/dL | 13.6 ± 1.9 | 13.1 ± 2.1 | <0.001 |
| TC, mg/dL | 178.4 ± 42.0 | 169.2 ± 38.1 | <0.001 |
| HDL-C, mg/dL | 51.3 ± 13.8 | 48.0 ± 13.3 | <0.001 |
| LDL-C, mg/dL | 99.2 ± 35.0 | 91.7 ± 31.0 | <0.001 |
| TG, mg/dL | 138.6 ± 101.1 | 143.0 ± 81.5 | 0.211 |
| eGFR, mL/min/1.73 m^2^ | 72.7 ± 33.6 | 64.9 ± 34.5 | <0.001 |
| cfPWV, carotid femoral pulse wave velocity; BMI, body mass index; SBP, systolic blood pressure; DBP, diastolic blood pressure; RASB, renin angiotensin system blockers; CCB, calcium channel blockers; BB, beta blockers; TC, total cholesterol; HDL-C, high-density lipoprotein cholesterol; LDL-C, low-density lipoprotein cholesterol; TG, triglyceride; BUN, blood urea nitrogen; eGFR, estimated glomerular filtration rate. | | | |

Supplemental Table 4. The clinical characteristics of study participants according to categories of central pulse pressure

|  | Central PP <49 mmHg  (n=2173) | Central PP ≥49 mmHg  (n=861) | P value |
| --- | --- | --- | --- |
| Age, years | 57.2 ± 11.7 | 64.1 ± 9.7 | <0.001 |
| Men, N(%) | 1248 (57.4) | 408 (47.4) | <0.001 |
| BMI, kg/m^2^ | 25.1 ± 3.7 | 25.0 ± 3.5 | 0.344 |
| Hypertension, N(%) | 1792 (82.5) | 743 (86.3) | 0.012 |
| Type 2 diabetes, N(%) | 908 (41.8) | 473 (54.9) | <0.001 |
| SBP, mmHg | 122.6 ± 14.0 | 142.5 ± 19.1 | <0.001 |
| DBP, mmHg | 76.4 ± 10.2 | 77.6 ± 11.7 | 0.010 |
| Antihypertensive medications, N(%) | 1754 (80.7) | 754 (87.6) | <0.001 |
| RASB, N(%) | 1376 (63.3) | 559 (64.9) | <0.001 |
| CCB, N(%) | 1002 (46.1) | 505 (58.7) | <0.001 |
| BB, N(%) | 479 (22.0) | 328 (38.1) | <0.001 |
| Diuretics, N(%) | 486 (22.4) | 269 (31.2) | <0.001 |
| Laboratory |  |  |  |
| Hemoglobin, g/dL | 13.7 ± 1.9 | 12.5 ± 2.0 | <0.001 |
| TC, mg/dL | 175.1 ± 41.2 | 169.3 ± 37.4 | <0.001 |
| HDL-C, mg/dL | 50.1 ± 13.5 | 48.0 ± 13.9 | <0.001 |
| LDL-C, mg/dL | 96.2 ± 33.7 | 92.7 ± 31.3 | 0.011 |
| TG, mg/dL | 143.6 ± 96.9 | 134.5 ± 73.7 | 0.008 |
| eGFR, mL/min/1.73 m^2^ | 72.5 ± 32.4 | 58.3 ± 36.7 | <0.001 |
| PP, pulse pressure; BMI, body mass index; SBP, systolic blood pressure; DBP, diastolic blood pressure; RASB, renin angiotensin system blockers; CCB, calcium channel blockers; BB, beta blockers; TC, total cholesterol; HDL-C, high-density lipoprotein cholesterol; LDL-C, low-density lipoprotein cholesterol; TG, triglyceride; BUN, blood urea nitrogen; eGFR, estimated glomerular filtration rate. | | | |

Supplementary Table 5. The association between arterial stiffness measures and incident heart failure in participants with ankle-brachial index ≥0.9 (N=2942).

|  | Univariable model | | Age- and sex-adjusted | | Multivariable model | |
| --- | --- | --- | --- | --- | --- | --- |
|  | HR (95% CI) | P value | HR (95% CI) | P value | HR (95% CI) | P value |
| Categorical variables |  |  |  |  |  |  |
| baPWV ≥1835 cm/s | 2.63 (1.50-4.62) | <0.001 | 2.61 (1.43-4.76) | 0.002 | 1.47 (0.77-2.78) | 0.239 |
| Brachial PP ≥55 mmHg | 2.96 (1.75-5.02) | <0.001 | 3.05 (1.77-5.26) | <0.001 | 1.63 (0.88-3.02) | 0.123 |
| cfPWV ≥8.8 m/s | 2.90 (1.59-5.27) | <0.001 | 2.98 (1.58-5.63) | <0.001 | 2.04 (1.03-4.06) | 0.042 |
| Central PP ≥49 mmHg | 4.55 (2.67-7.75) | <0.001 | 4.97 (2.83-8.72) | <0.001 | 3.21 (1.70-6.05) | 0.001 |
| Continuous variables^*^ |  |  |  |  |  |  |
| baPWV, cm/s | 1.19 (0.95-1.49) | 0.123 | 1.17 (0.91-1.50) | 0.231 | 1.21 (0.61-1.13) | 0.237 |
| Brachial PP, mmHg | 1.83 (1.52-2.21) | <0.001 | 1.86 (1.54-2.25) | <0.001 | 1.49 (1.17-1.90) | 0.001 |
| cfPWV, m/s | 1.52 (1.26-1.84) | <0.001 | 1.55 (1.26-1.90) | <0.001 | 1.25 (0.98-1.61) | 0.076 |
| Central PP, mmHg | 1.80 (1.52-2.14) | <0.001 | 1.85 (1.55-2.22) | <0.001 | 1.51 (1.20-1.89) | <0.001 |
| ^*^HR for incident HF expressed per standard deviation increment in each measure. SD for each measure were as follows: baPWV SD=304.4 cm/s, brachial PP SD=11.6 mmHg, cfPWV SD=2.4 m/s, central PP SD=13.9 mmHg.  Multivariable model adjusted for age, sex, body mass index, diabetes, mean blood pressure, hypertensive medication usage, hemoglobin level, and estimated glomerular filtration rate.  HR, hazard ratio; CI, confidence interval; baPWV, brachial ankle pulse wave velocity; cfPWV, carotid femoral pulse wave velocity; PP, pulse pressure; SD, standard deviation. | | | | | | |
